# Castration in Indiana

**Published:** 1907-10

**Authors:** 


### Abstracts and Selections.

---

CASTRATION IN INDIANA.—Dr. H. C. Sharp, physician to the Indiana State Reformatory, at Jeffersonville, Ind., has advised the *Clinique* that the following bill, to which attention was called editorially last May, has been passed and is a statute of the State of Indiana:

A bill for an act entitled "An Act to prevent procreation of confirmed criminals, idiots, imbeciles and rapists; providing that superintendents and boards of managers of institutions where such persons are confined shall have the authority and are empowered to appoint a committee of experts, consisting of two (2) physicians, to examine into the mental condition of such inmates."

Whereas, Heredity plays a most important part in the transmission of crime, idiocy and imbecility;

Therefore, Be it enacted by the General Assembly of the State of Indiana, that on and after the passage of this act it shall be compulsory for each and every institution in the State entrusted with the care of confirmed criminals, idiots, rapists and imbeciles, to appoint upon its staff, in addition to the regular institutional physician, two (2) skilled surgeons of recognized ability, whose duty it shall be, in conjunction with the chief physician of the institution, to examine the mental and physical condition of such inmates as are recommended by the institutional physician and board of managers. If, in the judgment of this committee of experts and the board of managers, procreation is inadvisable and there is no probability of improvement of the mental condition of the inmate, it shall be lawful for the surgeons to perform such operation for the prevention of procreation as shall be decided safest and most effective. But this operation shall not be performed except in cases that have been pronounced unimprovable, provided, that in no case shall the consultation fee be more than three dollars ($3) to each expert, to be paid out of the funds appropriated for the maintenance of such institution.—*The Clinique,* Chicago.

Indiana got ahead of Texas that time. A bill for the benefit of the rapist passed the Texas House last session unanimously, and would have passed the Senate had it been reached. It died on the calendar. She goes—next session.

